# Examining financial distress of the Vietnamese listed firms using accounting-based models

**DOI:** 10.1371/journal.pone.0284451

**Published:** 2023-05-23

**Authors:** Thao Tran, Ngoc Hong Nguyen, Binh Thien Le, Nam Thanh Vu, Duc Hong Vo

**Affiliations:** 1 International School of Business, University of Economics Ho Chi Minh City, Ho Chi Minh City, Vietnam; 2 Research Centre in Business, Economics & Resources, Ho Chi Minh City Open University, Ho Chi Minh City, Vietnam; Bucharest University of Economic Studies: Academia de Studii Economice din Bucuresti, ROMANIA

## Abstract

Financial distress is generally considered the most severe consequence for firms with poor financial performance. The emergence of the Covid-19 pandemic has adversely impacted the global business system and exacerbated the number of financially distressed firms in many countries. Only firms with strong financial fundamentals can survive extreme events such as the Covid-19 pandemic and the ongoing Russia-Ukraine conflict. Vietnam is no exception. However, studies examining financial distress using accounting-based indicators, particularly at the industry level, have largely been ignored in the Vietnamese context, particularly with the emergence of the Covid-19 pandemic. This study, therefore, comprehensively examines financial distress for 500 Vietnamese listed firms during the 2012–2021 period. Our study uses interest coverage and times-interest-earned ratios to proxy a firm’s financial distress. First, our findings confirm the validity of Altman’s Z”- score model in Vietnam only when the interest coverage ratio is used as a proxy for financial distress. Second, our empirical findings indicate that only four financial ratios, including *EBIT/Total Assets*, *Net Income/Total Assets*, *Total Liabilities/Total Assets*, and *Total Equity/Total Liabilities*, can be used in predicting financial distress in Vietnam. Third, our analysis at the industry level indicates that the "Construction & Real Estates" industry, a significant contributor to the national economy, exhibits the most significant risk exposure, particularly during the Covid-19 pandemic. Policy implications have emerged based on the findings from this study.

## 1. Introduction

Financial distress contains several situations in which a firm faces financial difficulties [1], whereas firm defaults when it fails to service its debt obligations [2]. Financial distress and firm default are closely related. If a firm cannot generate sufficient earnings to cover its financial obligations, it is considered financially distressed. When the financially distressed condition continues–in other words, a firm consistently lacks financial liquidity, and the firm will be likely to go bankrupt [3]. In this regard, many firms attempt to manipulate their financial information disclosure, causing a lack of transparency in financial reporting. However, there is evidence indicating that poor financial reporting quality is highly correlated with greater financial distress risk [4]. As such, financially distressed firms, instead of manipulating financial information, should adopt well-established liquidity management strategies to prevent bankruptcy [5]. For many decades, a large body of literature has emerged on firm default prediction and corporate financial distress because of the paramount importance of understanding and predicting firms’ financial conditions, especially during a turbulent economic period [6]. In particular, the Covid-19 pandemic became widespread worldwide and immediately affected the global economy and business system. It triggered waves of business failures in many industries across many countries globally. The pandemic also resulted in many financially distressed firms in many countries, causing widespread economic distress and significantly impacting the global economy [7].

Vietnam is no exception. The prolonged Covid-19 pandemic in 2021 exposed the nation to significant economic risk. A report from Vietnam’s General Statistics Office (GSO) indicates that, in the third quarter of 2021, Vietnam experienced its first-ever negative GDP growth since 2000. Although Vietnam ended the year 2021 with a positive GDP growth rate of 2.58 per cent, this growth rate was significantly lower than those of the past 22 years [8]. The GSO’s report further shows that the Covid-19 pandemic, with substantial strict lockdown and prolonged social distancing, adversely affected many existing businesses in Vietnam. Vietnam witnessed 119,800 enterprises closing their businesses permanently in 2021 (17.8 per cent higher than in 2020), while the total number of newly established firms in the market was around 160,000 (a 10.7 per cent decline from 2020).

Studies on understanding financial distress in Vietnam, however, are limited. As a result, the effects of the Covid-19 pandemic on the financial distress of Vietnam’s listed firms have largely been ignored in the existing literature, particularly at the industry level [9]. As such, this paper comprehensively examines the financial distress of Vietnamese listed firms from 2012 to 2021, focusing on the Covid-19 pandemic. An analysis of Vietnamese financial distress at different levels, including both country and firm levels, is crucial, especially since Vietnamese firms have now put great effort into recovering after the pandemic. Findings from this study offer perspectives to researchers, practitioners, and policymakers on firms’ financial health in Vietnam, a new emerging market in the Asia-Pacific region, so that they can respond promptly.

Our study contributes to the existing literature three-fold. First, we examine the validity of the highly regarded Altman (1983) Z”-score model in Vietnam [10]. Few studies on Vietnamese financial distress have been conducted [11–13], primarily using accounting-based models in recent years, including the current Covid-19 pandemic. As such, in this study, we exclusively focus on the accounting-based model since accounting data are more accessible for existing market businesses. Moreover, accounting indicators reflect the fundamentals of firms’ operations, financial performance, and position. In addition, despite various alternative models of financial distress prediction, the Z-score model still plays a critical role for policymakers and practitioners in examining the likelihood of financial distress of listed firms in Vietnam.

Second, our analysis identifies well-fitted predictors from an extended list of financial indicators found in the financial distress literature. We consider that selected variables can perform very well in selected countries but may not be appropriate to explain the financial distress in Vietnam [6, 14]. Thus, identifying appropriate financial distress predictors in the Vietnamese context allows for early detection and, as such, prevention of financial distress for Vietnamese firms, particularly during major shocks such as the Covid-19 pandemic.

Finally, a comprehensive study examining financial distress will only be complete with an understanding of different levels of financial distress across industries. The reason is that the effects of financial distress, especially during the Covid-19 pandemic, may be heterogeneous and vary significantly for different industries [15–17]. As such, our study provides insights into the financial distress of each Vietnamese industry, signaling risk exposure of various industries to the Governments and those in other emerging economies so that they can implement immediate and effective measures.

Following this introduction, the remaining of this paper is structured as follows. Section 2 reviews and synthesizes the literature on corporate financial distress. Section 3 presents a research design including the data, the variables, and the empirical approaches used in this article. The main findings are presented and discussed in section 4 of the paper, followed by the concluding remarks and policy implications in section 5.

## 2 Review of related literature

This review aims to shed light on the early and recent prominent studies on default risk and corporate financial distress. First, the review outlines the literature related to accounting-based models, market-based models, and other related models. Each of these models is briefly discussed in turn below.

In the early stage, accounting-based models were widely adopted to predict the probability of bankruptcy, such as the archetypal study of Beaver (1966), which employed accounting-based variables to predict the solvency of firms [18]. With an initial set of 79 financial ratios for failed and non-failed listed companies, the author discovered the best firm failure predictor: the cash flow-to-debt ratio. Altman (1968) introduced the distinct distress prediction model using multivariate discriminant analysis (MDA) a few years later [19]. The author’s initial sample comprised 66 U.S. manufacturing companies (33 failed- and 33 non-failed firms) with 22 financial ratios. The author argued that using a discriminant-ratio model can improve the accuracy of the prediction ability. The findings from the paper proposed the discriminant function, a combination of five variables (out of 22 ratios), which can be used to calculate the probability of bankruptcy. Altman (1983) later realized that most privately-owned companies have no available market data [10]. As such, the author developed the other two models. The first model is named the Z-score model, which was intended for private manufacturing firms. In this model, the market value of firm equity is substituted by the book value of firm equity. The second model, generally known as the Z"-score model, was developed for both public and private firms and manufacturing and non-manufacturing firms. The sales-to-total-assets ratio was no longer considered in this second model.

The accounting-based and the Altman Z-score models have been the foundation for the financial distress area of research. Numerous studies have used accounting-based models and the Altman Z-score as proxies for financial distress because of their popularity and easy-to-use feature. However, accounting-based models’ prediction capability using the MDA is criticized when the model is applied to samples with distinct characteristics compared to the original sample. Hence, many alternative models have been developed to address these potential biases and to overcome the restrictive assumptions of MDA. These alternative models, for instance, include the conditional logit model [20], the probit model [21], the discrete hazard logit model [22], the market-based model [23], the machine learning model [24], or the deep learning-based model [25].

On the other hand, researchers also predict corporate financial distress using market-based measures with the seminal study of Merton (1974) [26]. Inspired by bond pricing when bankruptcy probability is high, Merton developed an option pricing model for pricing corporate liabilities or any financial instrument. This model, also known as the contingent claim methodology, is the building block for the literature on firm default prediction. Vassalou and Xing (2004) used the Merton model (1974) to estimate the probability of default risk for individual firms and explore the linkage between such risk and equity returns [2]. The analysis considered that size and book-to-market affect the default risk. Bharath and Shumway (2008) investigated the predictive power of the Merton distance to default (D.D.) model [27]. The conclusions indicated that the Merton DD probability model is an insufficient statistic for determining default probability. Still, structural models such as the Merton model can be used to develop default prediction models for firms. Likewise, Friewald et al. (2014) used the structural model of Merton (1974) to examine the association between stock returns and credit risk [28]. Findings from this study substantiate the hypothesis of the relationship between risk premia on equity and credit instruments. More recently, Atif and Ali (2021) [29] and Jia and Li (2022) [30] hypothesized the relationship between financial distress and corporate governance in the United States and Australia. Due to the models’ predictive ability, these studies used the market-based distance to default (D.D.) model.

The hazard model has been confirmed to have superior predictive power. Bauer and Agarwal (2014) argued that the model considers the time-domain perspective (changing characteristics over time) and allows the performance of default prediction at different time horizons [31]. Shumway (2001) used the hazard model to predict firm default [32]. The author confirmed that this model could be simply estimated and interpreted. Similarly, Campbell et al. (2008) used the dynamic logit model with market variables to investigate the factors affecting firm failure and the pricing of highly distressed stocks. More recently, Cathcart et al. (2020) analyzed the differential effect of leverage on default risk using the discrete hazard model in the form of a multi-period logit [33].

Apart from developing the appropriate prediction models from accounting-based and market-based variables, recent studies also attempt to explore the determinants affecting the probability of financial distress. These determinants can be classified into the following categories: (i) firm-level determinants [4, 34–37], (ii) country-risk determinants [14, 38–40], and (iii) corporate governance determinants [33, 41, 42]. Besides, machine learning models have also been used to improve the model accuracy of financial distress [39, 43–46].

Our literature review indicates that empirical studies on corporate financial distress have been extensively investigated. The financial distress of listed firms in Vietnam has also attracted the attention of various scholars [11–14]. However, studies using accounting-based models for the Vietnamese market appear to be outdated. The accounting-based model, the first and fundamental model for understanding financial distress, has largely been ignored in the Vietnamese context. In addition, studies on financial distress for Vietnamese firms during the Covid-19 pandemic in 2020 and 2021 have not been conducted. These observations warrant our analysis with a focus on using the accounting-based model to examine the financial distress of Vietnam’s listed firms.

## 3 Research sample

### 3.1 Data

Data for this study is collected from the Thomson Reuters Eikon database. The initial dataset includes 755 Vietnamese listed firms on Ho Chi Minh Stock Exchange (HOSE) and Hanoi Stock Exchange (HNX). Listed firms in the financial sector are operating under different regulations. As such, financial firms are removed from our sample. Furthermore, firms with missing financial data for more than five years are also removed from the sample. Finally, we winsorize all financial variables at 1 per cent and 99 per cent for the entire dataset to deal with the potential outliers. Our final sample includes 5,000 firm-year observations corresponding with 500 listed companies for 2012–2021.

### 3.2 Variables

Table 1 provides the descriptions of all variables used in this paper. The independent variables are selected from previous literature, including 11 financial indicators and three proxies for the firm’s characteristics. The 11 financial indicators are classified into three distinct groups liquidity, profitability, and leverage.

**Table 1:** Descriptions of variables.

Variable and abbreviation			Definition
**Dependent variables**			
Financial distress	Y_1_	ICR	A dummy variable of "1" when the interest coverage ratio is below one (distressed) and "0" otherwise (non-distressed).
	Y_2_	TIE	A dummy variable of "1" when the times-interest-earned is below one (distressed) and "0" otherwise (non-distressed).
**Explanatory variables**			
Liquidity	X_1_	WC/TA	Working capital to total assets
	X_2_	CA/CL	Current assets to current liabilities
	X_3_	CA/TL	Current assets to total liabilities
	X_4_	CL/TA	Current liabilities to total assets
Profitability	X_5_	RE/TA	Retained earnings to total assets
	X_6_	EBIT/TA	Earnings before interest and taxes to total assets
	X_7_	SALES/TA	Total sales to total assets
	X_8_	NI/TA	Net income to total assets
Leverage	X_9_	TL/TA	Total liabilities to total assets
	X_10_	MVE/TL	The market value of equity to total liabilities
	X_11_	TE/TL	Book value of equity to total liabilities
Firm characteristics	X_12_	SIZE	Firm size (logarithm of total assets)
	X_13_	AGE	The number of years since the firm’s incorporation
	X_14_	INDUSTRY	Industry dummies (firms are categorized into 13 industries under the NAICS 2007 standard)

#### 3.2.1 Measure of financial distress

A firm is considered financially distressed if it cannot satisfy financial obligations in the short term [1, 4]. Asquith et al. (1994) examine the importance of financial debt redemption [47]. As such, when companies generate insufficient revenue to cover their financial obligations, they can be classified as financially distressed leading to bankruptcy. As such, a probability of financial distress should be identified via the difference between its earnings (EBIT or EBITDA) and its interest payments. Previous scholars employ two indicators, including (i) the interest coverage ratio (ICR)–the ratio between earnings before interest and taxes (EBIT) and interest payments [11, 14, 47]; and (ii) the times-interest-earned (TIE)–the ratio between earnings before interest, taxes, depreciation, and amortization (EBITDA) and interest payments [48–50].

This study used the above two financial distress likelihood indicators to define the state of a firm’s financial distress for each year during the 2012–2021 period. When a ratio for a particular firm is lower than one, then this firm is classified as "distressed", which is then assigned a value of “1”. The "safe" or "non-distressed" firm is assigned as “0”. Table 2 presents financial distress classification using the ICR and the TIE.

**Table 2:** Descriptive statistics of dependent variables.

Dependent variables		Frequency	Per cent
	0	3,626	83
The interest coverage ratio (ICR)	1	759	17
	**Total**	**4,385**	**100**
	0	3,934	90
The times-interest-earned (TIE)	1	451	10
	**Total**	**4,385**	**100**

#### 3.2.2 Candidate predictors

The independent variables are selected from previous studies on corporate financial distress and firm default risk [6, 19–21, 27, 32, 48, 49, 51]. Table 3 presents the descriptive statistics of the explanatory variables used in the paper. The correlation matrix for these variables is presented in Table A1 (S1 Appendix).

**Table 3:** Descriptive statistics of explanatory variables.

Variable		Obs.	Mean	Std. Dev.	Min	Max
WC/TA	Liquidity ratios	5,000	.22	.22	-.26	.81
CA/CL		5,000	2.36	2.73	.40	18.31
CA/TL		5,000	1.86	2.29	.14	15.77
CL/TA		5,000	.39	.213	.03	.88
RE/TA	Profitability ratios	5,000	.06	.11	-.44	.39
EBIT/TA		5,000	.07	.07	-.12	.34
SALES/TA		5,000	1.15	.98	.02	5.34
NI/TA		5,000	.06	.07	-.15	.30
TL/TA	Leverage ratios	5,000	.50	.22	.04	.92
MVE/TL		4,685	2.02	4.11	.04	28.90
TE/TL		5,000	2.11	3.63	.09	23.81
SIZE	Firm characteristics	5,000	7.49	.67	5.97	9.26
AGE		5,000	9.23	6.50	0	61
INDUSTRY		5,000	6.58	2.75	0	12

Notes: **WC/TA** (Working Capital / Total Assets); **CA/CL** (Current assets / Current Liabilities); **CA/TL** (Current assets / Total Liabilities); **CL/TA** (Current Liabilities / Total Assets); **RE/TA** (Retained Earnings / Total Assets); **EBIT/TA** (Earnings Before Interest and Taxes / Total Assets); **SALES/TA** (Sales / Total Assets); **NI/TA** (Net Income / Total Assets); **TL/TA** (Total Liabilities / Total Assets); **MVE/TL** (Market Value of Equity / Total Liabilities); **TE/TL** (Book Value of Equity / Total Liabilities); **SIZE** (Total Assets in Natural Logarithm); **AGE** (the number of years since a firm’s establishment); **INDUSTRY** (Industry dummies)

## 4 Empirical methodology

### 4.1 The Z”-score model

This section reviews the Z-score models utilized in the study. The literature review reveals that the original Altman Z-score [19] was first developed for publicly manufacturing firms. The modified versions were then developed in Altman’s (1983) study [10] for different types of enterprises. The Altman’s Z’-score was developed for private manufacturing firms, while the Altman’s Z"-score model was introduced for both publicly and private manufacturing and non-manufacturing firms. Later, Altman’s (2005) [52] emerging market score (EMS) model was introduced to estimate the default probability for emerging markets [10, 52]. In this study, we are interested in the Z"-score model [10] and EMS model [52] because these two models have used identical financial indicators in predicting financial distress and can be used for an emerging market like Vietnam. The only difference between the Z”-score model and the EMS model is the term +3.25, which is added when considering the emerging economies in the EMS model. The predictors and corresponding estimated coefficients from the Z"-score model are as follows.


$$Z"=6.56*X_{1}+3.26*X_{2}+6.72*X_{3}+1.05*X_{4}$$

The predictors and corresponding estimated coefficients from the EMS model are as follows.

$$EMS=6.56*X_{1}+3.26*X_{2}+6.72*X_{3}+1.05*X_{4}+3.25$$

where: X_1_ denotes the ratio between working capital and total assets (WC/TA); X_2_ represents the ratio between retained earnings and total assets (RE/TA); X_3_ denotes the ratio between earnings before interest and taxes and total asset (EBIT/TA); X_4_ represents the ratio between the book value of equity and the total liability (BVE/TL).

### 4.2 The analysis procedures

**Fig 1:**
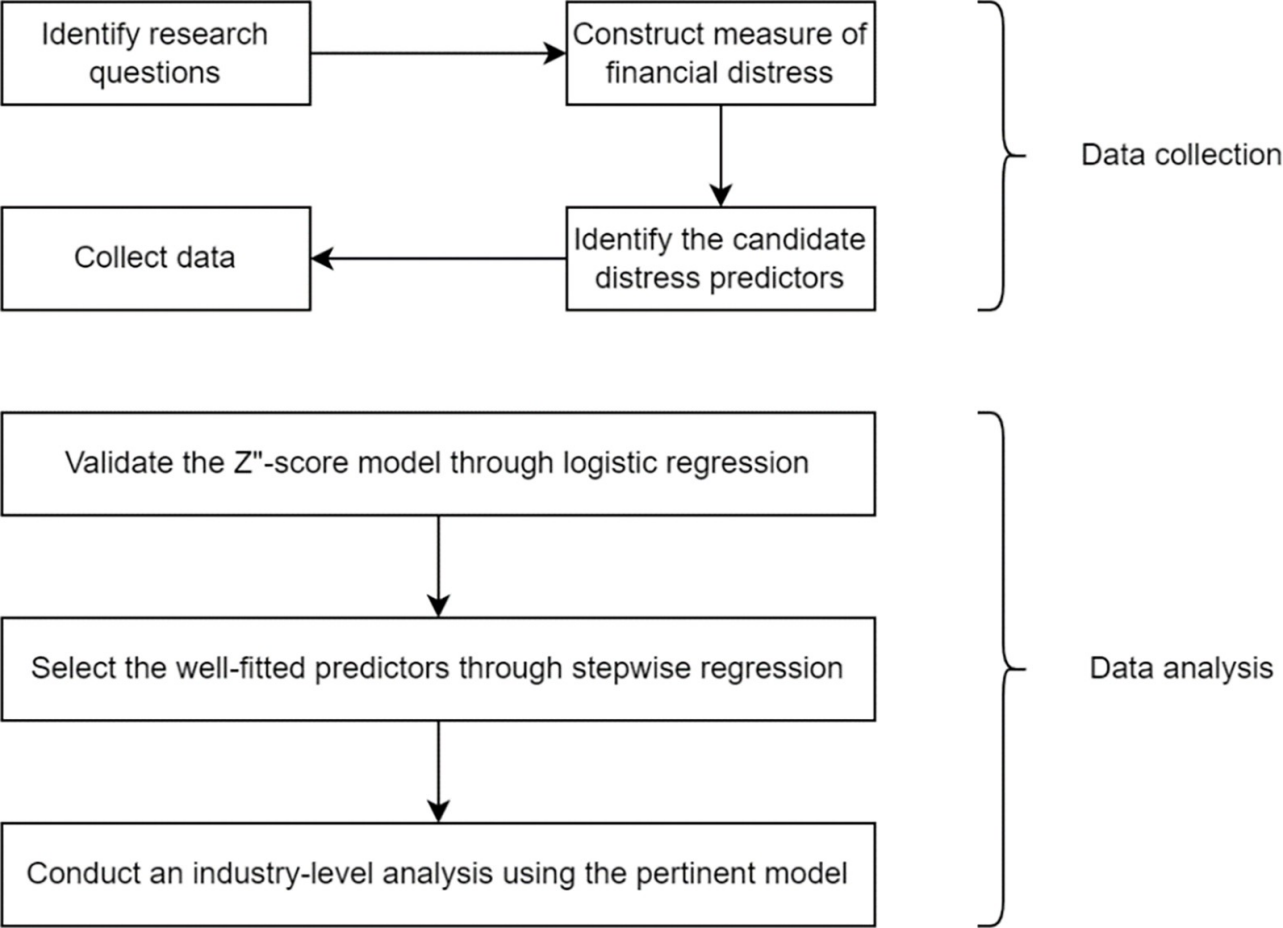
The step–by–step procedures used in the study’s empirical methodology.

This section briefly discusses the step-by-step procedure adopted in this study regarding the study’s empirical methodology. Fig 1 provides this step-by-step adopted procedure. *First*, we validate the Z”-score model, a well-regarded model for financial distress prediction, in the context of Vietnam. As such, we adopt the logistic regression and then estimate the area under the receiver operating characteristic curve (ROC) to measure the model predictive ability.

Regarding the logistic regression, previous studies indicate that this technique has outperformed the multi-discriminant analysis, especially for a large dataset. This is because the multi-discriminant analysis requires several assumptions that financial ratios analysis often cannot satisfy, whereas the logistic regression analysis requires no such restrictive assumptions. For this analysis, the distressed firms are assigned a value of 1, whereas 0 is assigned to non-distressed firms. Thus, with X_i_ are independent variables, and β_i_ are the estimated coefficients, the probability of financial distress for firms is as follows:

$$P(Y=1|X)=\frac{1}{1+e^{-L}}=\frac{1}{1+e^{-(\beta_{0}+\beta_{1}X_{1}+\beta_{2}X_{2}+\beta_{3}X_{3}+\beta_{4}X_{4})}}$$

Regarding the ROC, it is a powerful approach for model evaluation since the approach directly examines the predictive accuracy of the models [14, 53]. The ROC has a close connection with the accuracy ratio (A.R.), which is formulated as: A.R. = 2*(ROC—0.5). The AR of 1 indicates a perfect model, while the AR of 0.5 shows the average performance of the model. In contrast, the A.R. assigned a value of 0 signals a random model with no discriminatory power.

*Second*, we identify the accounting-based variables that can predict financial distress for Vietnamese listed firms. Hence, we adopt the stepwise regression to select the most pertinent distress predictors in the context of Vietnam. The potential predictors include 11 accounting-based variables which have been widely-used in the existing literature. The summary of these potential predictors used in the stepwise regression are presented in Table A2 (S1 Appendix). Efroymson (1960) was the first to introduce this technique [54]. The stepwise regression is conducted automatically to fit the regression models using selected variables. Each step of the procedure examines the relevance of each variable to determine its inclusion or removal based on a pre-specified criterion [55].

*Third*, a comprehensive analysis would be incomplete without an analysis at the industry-level. Therefore, we utilize the emerging-market score (EMS) model, which was discussed in the previous sub-section, to conduct the analysis at the industry level. In particular, we first compute the EMS scores for each firm for each year across the 10-year period. Firms are then classified in accordance with the following thresholds: *Firms with an EMS greater than 5*.*85 are classified as SAFE; firms with an EMS less than 4*.*15 are classified as DISTRESS; The remaining firms are classified as GREY*. Thereafter, based on the classification results, we calculate the number of firms for each above-mentioned category across industries for the whole period (2012–2021) and for the period of Covid-19 pandemic (2020–2020).

## 5 Empirical results

This section presents the main findings of our empirical analyses. First, the logit regression analysis is employed to validate the Altman Z”-score model. Second, the stepwise regression is used to identify well-fitted distress predictors for the Vietnamese listed firms. Finally, Altman’s (2005) [52] Emerging Market Score (EMS) model estimates the financial distress likelihood of Vietnamese firms in each industry.

### 5.1 The baseline regression

The original Z-score of Altman (1968) [19] and two modified versions, including Altman’s (1983) [10] Z’-score and Z"-score models, have been widely used in understanding the financial distress of firms. They are well-tested and straightforward. The Z"-score model has been re-assessed by Altman (2017) [6] using a sample of firms from international countries. Furthermore, accounting-based data is available for firms. Financial indicators from the Z”-score model are used to examine their appropriateness in predicting the financial distress likelihood for Vietnamese listed firms. Two financial distress indicators, including (i) the interest coverage ratio (ICR) and (ii) the times-interest-earned (TIE), are utilized in our analysis.

Table 4 presents empirical results from the logistic regression regarding the financial distress of Vietnamese firms from 2012 to 2021. Ten models are used. In addition, the area under the receiver operating characteristic curve (ROC) is used to examine the predictive ability of the models. Ten regression models are as follows:

**Table 4:** The financial distress of Vietnamese firms using both ICR and TIE as proxies for financial distress.

Model 1:	The ICR is used together with four Altman Z"-score variables (financial indicators).
Model 2:	The TIE is used together with four Altman Z"-score variables.
Model 3:	Model (1) with the firm’s size (*Size*).
Model 4:	Model (2) with the firm’s size (*Size*).
Model 5:	Model (1) with the firm’s age (*Age*).
Model 6:	Model (2) with the firm’s age (*Age*).
Model 7:	Model (1) with the industry dummies *(Industry)*.
Model 8:	Model (2) with the industry dummies *(Industry)*.
Model 9:	Model (1) with the firm’s size, firm age, and industry dummies.
Model 10:	Model (2) with the firm’s size, firm age, and industry dummies.

Model	(1)	(2)	(3)	(4)	(5)	(6)	(7)	(8)	(9)	(10)
Variable	ICR	TIE	ICR	TIE	ICR	TIE	ICR	TIE	ICR	TIE
WC/TA	**-3.00** ***	**-0.74** *	**-2.91** ***	-0.63	**-3.05** ***	**-0.82** **	**-3.05** ***	**-0.81** *	**-3.04** ***	**-0.83** *
	(-8.43)	(-1.90)	(-8.16)	(-1.62)	(-8.53)	(-2.11)	(-8.10)	(-1.91)	(-8.12)	(-1.94)
RE/TA	**-2.71** ***	-0.89	**-3.00** ***	-1.17	**-2.59** ***	-0.76	**-3.02** ***	-0.40	**-3.28** ***	-0.66
	(-3.03)	(-0.95)	(-3.32)	(-1.17)	(-2.91)	(-0.78)	(-3.20)	(-0.37)	(-3.41)	(-0.54)
EBIT/TA	**-117.81** ***	**-89.28** ***	**-117.94** ***	**-90.98** ***	**-118.01** ***	**-89.74** ***	**-124.48** ***	**-91.50** ***	**-125.22** ***	**-94.00** ***
	(-21.66)	(-12.86)	(-21.69)	(-12.68)	(-21.70)	(-12.96)	(-21.49)	(-12.60)	(-21.57)	(-12.59)
TE/TL	**-0.06** **	-0.02	**-0.05** *	-0.01	**-0.05** **	-0.02	**-0.09** ***	-0.01	**-0.08** **	0.01
	(-2.09)	(-0.86)	(-1.70)	(-0.32)	(-2.02)	(-0.70)	(-3.01)	(-0.46)	(-2.53)	(0.22)
SIZE			1.89	**5.89** ***					2.20	**6.82** ***
			(1.10)	(2.73)					(1.18)	(2.92)
SIZE^2			-0.11	**-0.36** ***					-0.13	**-0.42** ***
			(-1.00)	(-2.62)					(-1.05)	(-2.78)
AGE					-0.02	**-0.06** **			**-0.04** **	**-0.06** *
					(-1.55)	(-2.02)			(-2.43)	(-1.84)
**Industry**										
AWS							-0.55	-0.05	-0.21	0.43
							(-0.50)	(-0.06)	(-0.19)	(0.48)
AGP							0.96	0.33	0.93	0.60
							(1.00)	(0.37)	(0.95)	(0.63)
AER							-0.69	**-3.07** ***	-0.68	**-2.98** ***
							(-0.56)	(-3.28)	(-0.57)	(-3.02)
CRE							-0.14	0.10	-0.04	0.26
							(-0.17)	(0.15)	(-0.05)	(0.40)
IAT							-0.26	-0.20	0.14	0.49
							(-0.28)	(-0.26)	(0.16)	(0.64)
MNF							0.83	-0.60	1.01	-0.31
							(1.00)	(-0.90)	(1.23)	(-0.45)
MOG							0.32	-0.59	0.45	-0.27
							(0.37)	(-0.80)	(0.53)	(-0.36)
PST							-1.24	-0.00	-0.84	0.76
							(-1.19)	(-0.00)	(-0.81)	(0.77)
RTT							0.09	-0.07	0.29	0.28
							(0.10)	(-0.10)	(0.34)	(0.38)
TAW							1.03	-0.55	1.26	-0.24
							(1.14)	(-0.64)	(1.42)	(-0.28)
ULT							-0.45	-0.87	-0.40	-0.92
							(-0.50)	(-1.20)	(-0.45)	(-1.25)
WST							0.69	0.93	0.86	**1.19** *
							(0.83)	(1.35)	(1.04)	(1.70)
_cons	**2.13** ***	**-0.30** **	-5.82	**-23.97** ***	**2.34** ***	0.20	**2.06** **	-0.24	-7.09	**-27.56** ***
	(14.92)	(-2.13)	(-0.88)	(-2.87)	(11.99)	(0.77)	(2.52)	(-0.38)	(-0.98)	(-3.02)
*N*	4385	4385	4385	4385	4385	4385	4385	4385	4385	4385
pseudo *R*^2^	0.656	0.609	0.657	0.613	0.657	0.613	0.668	0.624	0.671	0.632
ROC curve	0.970	0.974	0.970	0.975	0.970	0.974	0.972	0.976	0.973	0.977

Notes: Standard errors in parentheses * p<0.1, ** p<0.05, *** p<0.01. **Model 1:** The ICR is used with four Altman Z"–score variables; **Model 2:** The TIE is used with four Altman Z"–score variables; **Model 3:** Model (1) and the firm’s size (Size); **Model 4:** Model (2) and the firm’s size (Size); **Model 5:** Model (1) and the firm’s age (Age); **Model 6:** Model (2) and the firm’s age (Age); **Model 7:** Model (1) and the industry dummies (Industry); **Model 8:** Model (2) and the industry dummies (Industry); **Model 9:** Model (1) and the firm’s size, firm’s age, and industry dummies; **Model 10:** Model (2) and the firm’s size, firm’s age, and industry dummies.

**AWS** for Administrative & Support Services; **AGP** for Agricultural Production; **AER** for Arts Entertainment, & Recreation; **CRE** for Construction & Real Estate; **IAT** for Information & Technology; **MNF** for Manufacturing; **MOG** for Mining, Quarrying, Oil, and Gas Extraction; **PST** for Professional, Scientific, & Technical Services; **RTT** for Retail trade; **TAW** for Transportation & Warehousing; **UTL** for Utilities; and **WST** for Wholesale Trade.

Table 4 presents empirical results. The estimated coefficients of the regressors used in Model 1 are statistically significant. These findings show that financial indicators used in the Altman Z"-score model can explain financial distress in Vietnam when the ICR is used as a proxy for financial distress. These findings indicate that the greater these financial ratios, the better the company’s performance, resulting lower probability of financial distress. Additionally, the estimated coefficient of the TE/TL ratio is relatively small, implying the small effect of this indicator on financial distress using the logit model. These findings are consistent with previous financial distress studies [6, 10, 12, 14]. Results from models 3, 5, 7, and 9 using the ICR as a proxy for financial distress show that a firm’s size, age, and industry effects do not generally contribute to a firm’s financial distress.

However, when the TIE has used a proxy for financial distress, findings from model 2 suggest that only two out of four estimated coefficients, WC/TA and EBIT/TA, can be used to predict financial distress for Vietnamese listed companies. Additionally, models 4, 6, 8, and 10 confirm the effect of a firm’s size and age on firms’ financial distress in Vietnam.

We now use results from the ROC, as presented in Table 4, to examine the performance of these models. All models can predict the financial distress likelihood for Vietnamese listed firms because of their statistical significance of the ROC (greater than 0.5). Based on the estimated values of the ROC, we note that models using the TIE as a proxy for the financial distress of listed firms in Vietnam perform slightly better than those using the ICR. Among them, the best-performing models are the comprehensive models (model 9 using the ICR and model 10 using the TIE). These two models show the highest ROC values of 0.973 (for ICR) and 0.977 (for TIE).

### 5.2 Examining and identifying well-fitted predictors for financial distress for listed firms in Vietnam

This section uses stepwise regression to identify well-fitted predictors from 11 widely used financial ratios in previous literature to examine firms’ financial distress. These financial indicators are presented in Table A2 (S1 Appendix). These ratios are classified into three categories: (i) Liquidity, (ii) Profitability, and (iii) Leverage. In addition, two financial distress indicators are used as proxies for financial distress, including the interest coverage ratio (ICR) and the times-interest-earned (TIE).

Table 5 presents empirical findings using the stepwise logistic regression for the full sample from 2012 to 2021. When the ICR is used as a proxy for financial distress, eight of 11 widely used indicators are considered appropriate for predicting the financial distress of Vietnamese listed firms. However, when the TIE is used as a proxy for financial distress, only four indicators are found appropriate for the purpose, including (i) X_6_ = EBIT/TA, and (ii) X_8_ = NI/TA (for *Profitability*); and (iii) X_9_ = TL/TA, and (iv) X_11_ = TETL (for *leverage*). No ratios on *Liquidity* are considered appropriate for examining the financial distress of Vietnamese listed firms.

**Table 5:** The stepwise logistic regression analysis for the full sample.

Proxy	Equation	ROC
ICR	$P(Y_{1}=1)=1.607-3.712X_{1}+0.307X_{3}-1.769X_{4}-123.127X_{6}+0.225X_{7}-18.146X_{8}+2.081X_{9}-0.155X_{11}$	0.9732
TIE	$P(Y_{2}=1)=-1.767-92.079X_{6}-6.007X_{8}+2.203X_{9}-0.084X_{11}$	0.9759

*Notes*: This table presents empirical results using logit regressions for Vietnamese listed firms during the 2012–2021 period, including groups of distressed firms and non–distressed firms using both ICR and TIE as proxies for the firm’s financial distress.

As a robustness analysis, our entire sample is now divided into four sub-samples, including (i) the pre-Covid-19 period (*2012–2019*); (ii) the Covid-19 period (*2020–2021*); (iii) firms listed in Ho Chi Minh City stock exchange only (*HOSE*); and (iv) firms listed in Ha Noi stock exchange only (*HNX*). Table 6 presents the summary of selected financial indicators using stepwise regressions across two different proxies for a firm’s financial distress. We note that the results remain largely similar to Table 5.

**Table 6:** A summary of selected variables in predicting financial distress across scenarios in Vietnam.

**Y**_**1**_ **is the dependent variable (Interest coverage ratio–ICR)**			**Full Sample**	**2012–2019**	**2020–2021**	**HOSE**	**HNX**
**Liquidity**	X_1_	WC/TA	x	x	x	x	x
	X_2_	CA/CL			x		
	X_3_	CA/TL	x	x		x	x
	X_4_	CL/TA	x	x		x	x
**Profitability**	X_5_	RE/TA		x			
	X_6_	EBIT/TA	x	x	x	x	x
	X_7_	SALES/TA	x	x	x	x	x
	X_8_	NI/TA	x	x	x	x	x
**Leverage**	X_9_	TL/TA	x	x	x	x	x
	X_10_	MVE/TL					
	X_11_	TE/TL	x	x		x	x
**Y**_**2**_ **is the dependent variable (Times-interest-earned–TIE)**			**Full Sample**	**2012–2019**	**2020–2021**	**HOSE**	**HNX**
**Liquidity**	X_1_	WC/TA					
	X_2_	CA/CL				x	x
	X_3_	CA/TL					
	X_4_	CL/TA					
**Profitability**	X_5_	RE/TA					
	X_6_	EBIT/TA	x	x	x	x	x
	X_7_	SALES/TA			x		
	X_8_	NI/TA	x	x	x	x	x
**Leverage**	X_9_	TL/TA	x	x	x	x	x
	X_10_	MVE/TL					
	X_11_	TE/TL	x	x	x	x	x

Financial indicators from the profitability ratios are dominant, followed by leverage ratios and Liquidity. Interestingly, while ratios from the liquidity group are dominant when the ICR is used as a proxy for financial distress, ratios of this group are found inappropriate to explain financial distress when the TIE is used as a proxy for financial distress. As presented in Table 6, two ratios in profitability (EBIT/TA and NI/TA) perform best across different financial distress proxies. The second-best performing ratios come from the leverage group (TL/TA and TE/TL).

### 5.3 The industry-level analysis

Examining firms’ financial distress at the industry level is important to capture the complete picture of corporate financial distress for listed firms in Vietnam because each industry is exposed to different levels of risk. This paper categorizes listed firms into 13 industries using the North American Industry Classification System (NAICS). The emerging-market score (EMS) model is then employed to estimate the financial distress likelihood for each industry.

Table 7 presents the inclusive EMS values (or financial distress likelihood) for each industry spanning over two periods: (i) from 2012 to 2021 (the entire period) and (ii) from 2020 to 2021 (the Covid-19 pandemic period). For the entire period, "Administrative & Support Services" appears to be the riskiest industry with a financial distress likelihood of 65 per cent (including 46 per cent of several firms classified as "grey" and 19 per cent as "distressed"). Other relatively high-risk industries include "Accommodation & Food Services" and "Construction & Real Estate". In contrast, "Arts, Entertainment, & Recreation" is the least risky exposure industry. It is noteworthy that the effects of the Covid-19 pandemic on a firm’s financial distress vary across Vietnamese industries. "Administrative & Support Services" becomes the second-lowest risk exposure during the pandemic period (from 2020 to 2021). Meanwhile, "Accommodation & Food Services" and "Construction & Real Estate" exhibit an upward trend in the number of financially distressed firms.

**Table 7:** The overall EMS estimates for each industry.

Industry name	Numbers of firms	2012–2021 (Per cent of firms)			2020–2021 (Per cent of firms)		
		Safe	Grey	Distressed	Safe	Grey	Distressed
Accommodation & Food Services	6	41	21	38	42	8	50
Administrative & Support Services	2	35	46	19	75	25	0
Agriculture Production	5	63	20	17	60	20	20
Arts Entertainment & Recreation	2	82	0	18	100	0	0
Construction & Real Estate	130	41	40	19	35	43	22
Information & Technology	24	76	17	7	69	25	6
Manufacturing	169	60	29	11	61	28	11
Mining, Quarrying, Oil, & Gas Extraction	26	54	17	29	56	17	27
Professional, Scientific, & Technical Services	8	63	27	10	63	31	6
Retail trade	17	52	30	19	62	26	12
Transportation & Warehousing	41	69	20	11	71	15	15
Utilities	31	51	32	17	58	31	11
Wholesale Trade	39	51	32	17	45	42	13

Notes: Listed firms are classified into industries under the NAICS 2007 (The North American Industry Classification System).

### 5.4 Discussion

Financial distress is probably one of the most vexing issues for a business. Much research is devoted to investigating corporate financial distress. Our study also contributes to the extant literature with a comprehensive analysis on corporate distress for Vietnam. *First*, we re-assess the Z”-score in the context of Vietnam because the model is well-known for its predictive performance. Empirical findings show that the model performs well in the Vietnamese market, but the results may vary regarding the different distress measures. Our results are in line with those of the existing literature, for instance, [6, 14, 37]. Further studies, however, could extend our study by validating the Z”-score model in the context of bankruptcy firms (firms that no longer exist in the market) instead of distressed firms–the focus in this study. *Second*, our study finds the most pertinent distress predictors for Vietnamese listed firms. The findings reveal that most of the well-fitted distress predictors are profitability ratios, implying that profitability seems to account for a substantial proportion of the financial distress risk of firms in the emerging market like Vietnam. Nevertheless, our study considers a set of traditional accounting-based determinants as potential financial distress predictors. Further research, therefore, should consider other types of distress determinants such as country risk factors or corporate governance to examine the accuracy of the models when these determinants are incorporated. *Third*, this study also provides an analysis at industry-level during the Covid-19 pandemic and shows that the number of distressed firms indeed increases in some certain industries. This finding, thus, helps practitioners, policymakers and alike to take the most appropriate response actions. In this regard, further studies could expand the sample coverage by considering a group of countries such as ASEAN countries or the emerging economies to draw more generalized and insightful conclusions.

## 6 Conclusions and policy implications

Financial distress is probably one of the most significant concerns for firms in Vietnam. The emergence of the Covid-19 pandemic has adversely affected the global business system and exacerbated the number of financially distressed firms worldwide. Vietnam is no exception. The country has witnessed an increasing number of closed businesses during the pandemic (2020 and 2021). Limited studies have examined firms’ financial distress in Vietnam, especially during the Covid-19 pandemic. Specifically, this paper extends previous studies by comprehensively investigating the financial distress of 500 Vietnamese listed firms from 2012 to 2021 using accounting-based variables. Financial distress is proxied by the interest coverage ratio (ICR); and the times-interest-earned (TIE). Key findings from the analyses can be summarized as follows.

First, we assess the validity of the highly regarded Altman (1983) [10] Z"-score model in the Vietnamese context. We find that when the ICR is used as a proxy for the financial distress likelihood of Vietnamese listed firms, the Altman Z"-score model performs satisfactorily in the Vietnamese context. However, when the TIE is used, only two of four financial indicators (including Working Capital/Total Assets and EBIT/Total Assets) in the model can predict the financial distress of Vietnamese listed firms. Second, we identify the well-fitted financial indicators for predicting financial distress for Vietnamese listed firms from 11 widely used financial ratios in three groups of financial ratios: (i) Liquidity, (ii) Profitability, and (iii) Leverage. On balance, well-fitted financial indicators in the Vietnamese context include EBIT/Total Assets and Net Income/Total Assets (for *Profitability* ratios) and Total Liabilities/Total Assets and Total Equity/Total Liabilities (for *Leverage* ratios). In contrast, Retained Earnings/Total Assets and Market value of Equity/Total Liabilities are found to be inappropriate for predicting financial distress for Vietnamese firms. Third, our extended analysis at the industry level reveals that "Arts, Entertainment, & Recreation" is the least risky exposure industry in contrast with the relatively high-risk industries of "Administrative & Support Services" and "Construction & Real Estate." Our findings also confirm the effects of the Covid-19 pandemic on firms’ financial distress across Vietnamese industries. The most significant aspect of our industry-based analysis is that the number of financially distressed firms had increased in "Construction & Real Estate" but decreased in "Administrative & Support Services" during the pandemic of 2020 and 2021. A complete lockdown of the Vietnamese economy in 2020, particularly during the lockdown of Ho Chi Minh City during the June–September 2021 period, the economic and financial centre of Vietnam, resulted in a significant increase in risk exposure for construction and real estate activities.

In summary, the Altman (1983) [10] Z”-score model performs well in examining financial distress for the Vietnamese listed firms when the interest coverage ratio (ICR) is used as a proxy for corporate financial distress. The ICR should be considered the best proxy for financial distress in Vietnam in understanding firms’ financial distress. In addition, our study provides evidence to support the use of EBIT/Total Assets, Net Income/Total Assets, Total Liabilities/Total Assets, and Total Equity/Total Liabilities in examining the financial distress of Vietnamese listed firms due to their well-fitted prediction power. Furthermore, our industry-level analysis also offers practitioners and policymakers valuable evidence regarding the risk of each industry. Immediate and practical support can be arranged to prevent the contagion effects from risky industries such as "Construction & Real Estate" to the entire system, especially during a turbulent period of the Covid-19 pandemic.

The contributions of our study to the existing literature on firms’ financial distress are threefold. First, we examine the validity of the Altman (1983) [10] Z”-score model in the context of Vietnam because there is a lack of empirical studies investigating financial distress in Vietnam, especially during the Covid-19 pandemic. Second, we identify the well-fitted financial distress indicators for Vietnamese-listed firms. Although numerous studies suggest a wide range of financial indicators for financial distress, these indicators may only be appropriate for other emerging markets, not Vietnam. Our findings, therefore, enable early detection of financial distress among Vietnamese listed firms, particularly during major shocks such as the Covid-19 pandemic. Third, we conduct the industry-level analysis focusing on the Covid-19 pandemic to fully capture the picture of Vietnamese financial distress at the industry level. Our findings confirm that the industry-level effects of financial distress are heterogenous and have varied significantly during the pandemic.

Besides, our findings also provide broader implications for other emerging economies sharing the similar characteristics of the Vietnamese market. Our study’s findings are useful in the sense of increased distressed firms and the downward trend in firms’ health among emerging markets in recent years. Stringent social distancing measures have exacerbated the number of closed businesses in many emerging countries. In addition, there is increased concern about the deterioration of firms’ health among emerging nations. Particularly, the proportion of liability in troubled firms in emerging nations is the greatest in the past decade. Our study denotes a single country analysis for a short period. Our findings can be generalized for other markets which still needs more back-up empirical evidence. As such, further comprehensive research on financial distress for the emerging markets is of the utmost importance, especially during a turbulent period like the Covid-19 pandemic accelerating the number of closed businesses. Further studies, thus, can improve this study by considering a sample of several emerging markets spanning a longer time period. Additional machine learning techniques and analysis procedures could also be used to draw more robust and insightful conclusions. In addition, further studies could consider incorporating other distress determinants such as real earning management, corporate social responsibility disclosures, or corporate governance variables.

## Supporting information

S1 Appendix (DOCX)

## References

[1] Geng R, Bose I, Chen X. Prediction of financial distress: An empirical study of listed Chinese companies using data mining. Eur J Oper Res. 2015 Feb;241(1):236–47.

[2] Vassalou M, Xing Y, Campbell J, Cochrane J, Chen L, French K, et al. Default Risk in Equity Returns. J Finance [Internet]. 2004 Apr 1 [cited 2023 Jan 28];59(2):831–68. Available from: https://onlinelibrary.wiley.com/doi/full/10.1111/j.1540-6261.2004.00650.x

[3] Zimon G, Nakonieczny J, Chudy-Laskowska K, Wójcik-Jurkiewicz M, Kochański K. An Analysis of the Financial Liquidity Management Strategy in Construction Companies Operating in the Podkarpackie Province. Risks. 2021 Dec 29;10(1):5.

[4] Tarighi H, Hosseiny ZN, Abbaszadeh MR, Zimon G, Haghighat D. How Do Financial Distress Risk and Related Party Transactions Affect Financial Reporting Quality? Empirical Evidence from Iran. Risks. 2022 Feb 23;10(3):46.

[5] Zimon G. Financial liquidity management strategies in polish energy companies. International Journal of Energy Economics and Policy. 2020 Mar 15;10(3):365–8.

[6] Altman EI, Iwanicz-Drozdowska M, Laitinen EK, Suvas A. Financial Distress Prediction in an International Context: A Review and Empirical Analysis of Altman’s Z-score Model. Journal of International Financial Management & Accounting. 2017 Jun;28(2):131–71.

[7] Amankwah-Amoah J, Khan Z, Wood G. COVID-19 and business failures: The paradoxes of experience, scale, and scope for theory and practice. European Management Journal. 2021 Apr;39(2):179–84. 10.1016/j.emj.2020.09.002 PMC7474582 38620607

[8] World Bank. Overview: Development news, research, data. The World Bank in Vietnam. 2022.

[9] Nguyen NH, Vu NT, Tran Q, Tran T, Vo DH. Market performance and volatility during the Covid-19 pandemic in Vietnam: A sector-based analysis. Cogent Business & Management [Internet]. 2022 [cited 2023 Jan 28];9(1). Available from: https://www.tandfonline.com/doi/abs/10.1080/23311975.2022.2119681

[10] Altman EI. Corporate financial distress: A complete guide to predicting, avoiding, and dealing with bankruptcy [Internet]. Vol. 5, Journal of Business Strategy. Wiley Interscience, John Wiley and Sons; 1983 [cited 2023 Jan 27]. Available from: http://www.loc.gov/catdir/enhancements/fy0607/82016103-b.html%5Cnhttp://www.loc.gov/catdir/enhancements/fy0607/82016103-d.html%5Cnhttp://www.loc.gov/catdir/enhancements/fy0607/82016103-t.html

[11] Dinh D V., Powell RJ, Vo DH. Forecasting corporate financial distress in the Southeast Asian countries: A market-based approach. J Asian Econ. 2021 Jun 1;74:101293.

[12] Vo DH, Pham BNV, Ho CM, McAleer M. Corporate Financial Distress of Industry Level Listings in Vietnam. Journal of Risk and Financial Management 2019, Vol 12, Page 155 [Internet]. 2019 Sep 22 [cited 2022 Jun 5];12(4):155. Available from: https://www.mdpi.com/1911-8074/12/4/155/htm

[13] Truong KD. Corporate governance and financial distress: An endogenous switching regression model approach in Vietnam. Cogent Economics & Finance. 2022 Dec 31;10(1).

[14] Pham B, Do T, Vo D. Financial distress and bankruptcy prediction: An appropriate model for listed firms in Vietnam. Economic Systems. 2018 Dec 1;42(4):616–24.

[15] Li C, Lou C, Luo D, Xing K. Chinese corporate distress prediction using LASSO: The role of earnings management. International Review of Financial Analysis. 2021 Jul;76:101776.

[16] Ugur M, Solomon E, Zeynalov A. Leverage, competition and financial distress hazard: Implications for capital structure in the presence of agency costs. Econ Model. 2022 Mar 1;108.

[17] Crespí-Cladera R, Martín-Oliver A, Pascual-Fuster B. Financial distress in the hospitality industry during the Covid-19 disaster. Tour Manag. 2021 Aug;85:104301.

[18] Beaver WH. Financial Ratios as Predictors of Failure. Journal of Accounting Research. 1966;4:71.

[19] Altman EI. Financial Ratios, Discriminant Analysis and the Prediction of Corporate Bankruptcy. J Finance. 1968 Sep;23(4):589.

[20] Ohlson JA. Financial Ratios and the Probabilistic Prediction of Bankruptcy. Journal of Accounting Research. 1980;18(1):109.

[21] Zmijewski ME. Methodological Issues Related to the Estimation of Financial Distress Prediction Models. Journal of Accounting Research. 1984;22:59.

[22] Charalambakis EC, Garrett I. On corporate financial distress prediction: What can we learn from private firms in a developing economy? Evidence from Greece. Review of Quantitative Finance and Accounting [Internet]. 2019 Feb 15 [cited 2023 Jan 27];52(2):467–91. Available from: https://link.springer.com/article/10.1007/s11156-018-0716-7

[23] Zhang X, Zhao Y, Yao X, Zhang X, Zhao Y, Yao X. Forecasting corporate default risk in China. Int J Forecast [Internet]. 2021 [cited 2022 Jun 6]; Available from: 10.1016/j.ijforecast.2021.04.009.

[24] Qian H, Wang B, Yuan M, Gao S, Song Y. Financial distress prediction using a corrected feature selection measure and gradient boosted decision tree. Expert Syst Appl. 2022 Mar;190:116202.

[25] Li S, Shi W, Wang J, Zhou H. A Deep Learning-Based Approach to Constructing a Domain Sentiment Lexicon: a Case Study in Financial Distress Prediction. Inf Process Manag. 2021 Sep;58(5):102673.

[26] Merton RC. On the Pricing of Corporate Debt: The Risk Structure of Interest Rates. J Finance. 1974 May;29(2):449.

[27] Bharath ST, Shumway T. Forecasting Default with the Merton Distance to Default Model. Rev Financ Stud [Internet]. 2008 May 1 [cited 2023 Jan 28];21(3):1339–69. Available from: https://academic.oup.com/rfs/article/21/3/1339/1566804

[28] Friewald N, Wagner C, Zechner J. The Cross-Section of Credit Risk Premia and Equity Returns. J Finance. 2014 Dec;69(6):2419–69.

[29] Atif M, Ali S. Environmental, social and governance disclosure and default risk. Bus Strategy Environ. 2021 Dec 14;30(8):3937–59.

[30] Jia J, Li Z. Corporate Environmental Performance and Financial Distress: Evidence from Australia. Australian Accounting Review. 2022 Jun 22;32(2):188–200.

[31] Bauer J, Agarwal V. Are hazard models superior to traditional bankruptcy prediction approaches? A comprehensive test. J Bank Financ. 2014 Mar 1;40(1):432–42.

[32] Shumway T. Forecasting bankruptcy more accurately: A simple hazard model. Journal of Business. 2001 Jan;74(1):101–24.

[33] Cathcart L, Dufour A, Rossi L, Varotto S. The differential impact of leverage on the default risk of small and large firms. Journal of Corporate Finance. 2020 Feb;60:101541.

[34] Valášková K, Adamko P, Michalikova KF, Macek J. Quo Vadis, earnings management? Analysis of manipulation determinants in Central European environment. Oeconomia Copernicana. 2021;12(3).

[35] Mitan A, Siekelova A, Rusu M, Rovnak M. Value-based management: a case study of visegrad four countries. Ekonomicko-manazerske spektrum. 2021;12(2):87–98.

[36] Kováčová M, Hrosova L, Durana P, Horak J. Earnings management model for Visegrad Group as an immanent part of creative accounting. Oeconomia Copernicana. 2022 Dec 30;13(4):1143–76.

[37] Liu L, Luo D, Han L. Default risk, state ownership and the cross-section of stock returns: evidence from China. Review of Quantitative Finance and Accounting. 2019 Nov 1;53(4):933–66.

[38] Tijani AA, Osagie RO, Afolabi BK. Effect of strategic alliance and partnership on the survival of MSMEs post COVID-19 pandemic. Ekonomicko-manazerske spektrum. 2021;15(2):126–37.

[39] Durana P, Perkins N, Valaskova K. Artificial Intelligence Data-driven Internet of Things Systems, Real-Time Process Monitoring, and Sustainable Industrial Value Creation in Smart Networked Factories. Journal of Self-Governance and Management Economics. 2021;9(2):21.

[40] Durana P, Michalkova L, Přívara A, Marousek J, Tumpach M. Does the life cycle affect earnings management and bankruptcy? Oeconomia Copernicana. 2021;12(2):425–61.

[41] Tao Q, Zahid Z, Mughal A, Shahzad F. Does operating leverage increase firm’s profitability and bankruptcy risk? Evidence from China’s entry into WTO. International Journal of Finance & Economics [Internet]. 2022 Oct 1 [cited 2023 Jan 28];27(4):4705–21. Available from: https://onlinelibrary.wiley.com/doi/full/10.1002/ijfe.2395

[42] García CJ, Herrero B. Female directors, capital structure, and financial distress. J Bus Res. 2021 Nov 1;136:592–601.

[43] Son H, Hyun C, Phan D, Hwang HJ. Data analytic approach for bankruptcy prediction. Expert Syst Appl. 2019 Dec;138:112816.

[44] Valaskova K, Ward P, Svabova L. Deep Learning-assisted Smart Process Planning, Cognitive Automation, and Industrial Big Data Analytics in Sustainable Cyber-Physical Production Systems. Journal of Self-Governance and Management Economics. 2021;9(2):9.

[45] Sun J, Fujita H, Zheng Y, Ai W. Multi-class financial distress prediction based on support vector machines integrated with the decomposition and fusion methods. Inf Sci (N Y). 2021 Jun;559:153–70. doi: 10.1016/j.ins.2021.01.059

[46] Xu J, Liu F. The Impact of Intellectual Capital on Firm Performance: A Modified and Extended VAIC Model. Journal of Competitiveness. 2020 Mar 31;12(1):161–76. doi: 10.7441/joc.2010.01.10

[47] Asquith P, Gertner R, Scharfstein D. Anatomy of Financial Distress: An Examination of Junk-Bond Issuers. Q J Econ [Internet]. 1994 Aug 1 [cited 2023 Jan 23];109(3):625–58. Available from: https://academic.oup.com/qje/article/109/3/625/1838275

[48] Pindado J, Rodrigues L, de la Torre C. Estimating financial distress likelihood. J Bus Res. 2008 Sep 1;61(9):995–1003.

[49] Tinoco MH, Wilson N. Financial distress and bankruptcy prediction among listed companies using accounting, market and macroeconomic variables. International Review of Financial Analysis. 2013 Dec 1;30:394–419.

[50] Konstantaras K, Siriopoulos C. Estimating financial distress with a dynamic model: Evidence from family owned enterprises in a small open economy. Journal of Multinational Financial Management. 2011 Oct;21(4):239–55.

[51] Deakin EB. A Discriminant Analysis of Predictors of Business Failure. Journal of Accounting Research. 1972 Spring;10(1):167.

[52] Altman EI. An emerging market credit scoring system for corporate bonds. Emerging Markets Review. 2005 Dec;6(4):311–23.

[53] Agarwal V, Taffler R. Comparing the performance of market-based and accounting-based bankruptcy prediction models. J Bank Financ. 2008 Aug;32(8):1541–51.

[54] Efroymson MA. Multiple regression analysis. Mathematical methods for digital computers. 1960;191–203.

[55] Draper NR, Smith H. Applied regression analysis. 1st ed. Vol. 326. John Wiley & Sons.; 1998.

