# Supplementary material for: Examining financial distress of the Vietnamese listed firms using accounting-based models
Source: PLoS One. 2023 May 23;18(5):e0284451. doi: 10.1371/journal.pone.0284451 (PMC10204956; doi:10.1371/journal.pone.0284451)
Supplement: S1 Appendix — (DOCX) [file pone.0284451.s001.docx]

# Appendix

**Table A1.** The correlation matrix of explanatory variables

| **Variable** | (X1) | (X2) | (X3) | (X4) | (X5) | (X6) | (X7) | (X8) | (X9) | (X10) | (X11) | (X12) | (X13) | (X14) |
| --- | --- | --- | --- | --- | --- | --- | --- | --- | --- | --- | --- | --- | --- | --- |
| (X1) WC/TA | 1.000 |  |  |  |  |  |  |  |  |  |  |  |  |  |
| (X2) CA/CL | 0.634* | 1.000 |  |  |  |  |  |  |  |  |  |  |  |  |
| (X3) CA/TL | 0.632* | 0.896* | 1.000 |  |  |  |  |  |  |  |  |  |  |  |
| (X4) CL/TA | -0.449* | -0.544* | -0.429* | 1.000 |  |  |  |  |  |  |  |  |  |  |
| (X5) RE/TA | 0.315* | 0.195* | 0.200* | -0.265* | 1.000 |  |  |  |  |  |  |  |  |  |
| (X6) EBIT/TA | 0.174* | 0.062* | 0.106* | -0.170* | 0.576* | 1.000 |  |  |  |  |  |  |  |  |
| (X7) SALES/TA | 0.011* | -0.132* | -0.034* | 0.232* | 0.149* | 0.274* | 1.000 |  |  |  |  |  |  |  |
| (X8) NI/TA | 0.330* | 0.203* | 0.237* | -0.314* | 0.669* | 0.875* | 0.225* | 1.000 |  |  |  |  |  |  |
| (X9) TL/TA | -0.589* | -0.581* | -0.620* | 0.781* | -0.310* | -0.245* | 0.015 | -0.407* | 1.000 |  |  |  |  |  |
| (X10) MVE/TL | 0.396* | 0.721 | 0.778* | -0.461* | 0.250* | 0.284* | -0.067* | 0.388* | -0.580* | 1.000 |  |  |  |  |
| (X11) TE/TL | 0.440* | 0.832* | 0.894* | -0.552* | 0.150* | 0.082* | -0.115* | 0.209* | -0.694* | 0.802* | 1.000 |  |  |  |
| (X12) SIZE | -0.254* | -0.223* | -0.311* | 0.095* | 0.100* | 0.000 | -0.225* | -0.032* | 0.371* | -0.171* | -0.281* | 1.000 |  |  |
| (X13) AGE | -0.057* | -0.039* | -0.021 | 0.062* | 0.035* | 0.009 | 0.036* | 0.008 | 0.047* | 0.028* | -0.029* | 0.143* | 1.000 |  |
| (X14) INDUSTRY | -0.017 | 0.080* | 0.080* | -0.160* | 0.104* | 0.107* | 0.310* | 0.135* | -0.179* | 0.095* | 0.129* | -0.061* | 0.058* | 1.000* |

Note: * represents a 5 per cent level of significance.

**Table A2.** Lists of utilized financial indicators for stepwise regression

| **Variable and abbreviation** | | | **Definition** |  |
| --- | --- | --- | --- | --- |
| **Dependent variables** | | | |  |
| Financial distress | Y_1_ | ICR | A dummy variable of "1" when the interest coverage ratio is below one (distressed) and "0" otherwise (non-distressed). | |
|  | Y_2_ | TIE | A dummy variable of "1" when the times-interest-earned is below one (distressed) and "0" otherwise (non-distressed). | |
| **Explanatory variables** | | | |  |
| Liquidity | X_1_ | WC/TA | Working capital to total assets | Altman (1968), Deakin (1972), Ohlson (1980), Shumway (2001) |
|  | X_2_ | CA/CL | Current assets to current liabilities | Ohlson (1980), Zmijewski (1984), Shumway (2001) |
|  | X_3_ | CA/TL | Current assets to total liabilities | Taffler (1983), Cathcart et al. (2020) |
|  | X_4_ | CL/TA | Current liabilities to total assets | Taffler (1983), Tinoco & Wilson (2013) |
| Profitability | X_5_ | RE/TA | Retained earnings to total assets | Altman (1968), Shumway (2001) |
|  | X_6_ | EBIT/TA | Earnings before interest and taxes to total assets | Altman (1968), Shumway (2001) |
|  | X_7_ | SALES/TA | Total sales to total assets | Altman (1968), Ohlson (1980), Shumway (2001) |
|  | X_8_ | NI/TA | Net income to total assets | Deakin (1972), Ohlson (1980), Zmijewski (1984), Shumway (2001) |
| Leverage | X_9_ | TL/TA | Total liabilities to total assets | Ohlson (1980), Zmijewski (1984), Shumway (2001), Tinoco & Wilson (2013) |
|  | X_10_ | MVE/TL | The market value of equity to total liabilities | Altman (1968) |
|  | X_11_ | TE/TL | Book value of equity to total liabilities | Altman (1983) |
